# Impact of *Clostridioides difficile* Infection on Clinical Outcomes in Hospitalized IBD Patients and the Role of Fecal Microbiota Transplantation: A Retrospective Cohort Study

**DOI:** 10.1002/kjm2.70002

**Published:** 2025-03-17

**Authors:** Puo‐Hsien Le, Chyi‐Liang Chen, Chia‐Jung Kuo, Pai‐Jui Yeh, Chien‐Chang Chen, Yi‐Ching Chen, Cheng‐Tang Chiu, Hao‐Tsai Cheng, Yung‐Kuan Tsou, Yu‐Bin Pan, Cheng‐Hsun Chiu

**Affiliations:** ^1^ Department of Gastroenterology and Hepatology Chang Gung Memorial Hospital Taoyuan Linkou Taiwan; ^2^ Chang Gung Inflammatory Bowel Disease Center Chang Gung Memorial Hospital Taoyuan Linkou Taiwan; ^3^ Chang Gung Microbiota Therapy Center Chang Gung Memorial Hospital Taoyuan Linkou Taiwan; ^4^ Taiwan Association for the Study of Intestinal Diseases (TASID) Taoyuan Taiwan; ^5^ Molecular Infectious Disease Research Center Chang Gung Memorial Hospital Taoyuan Linkou Taiwan; ^6^ Department of Pediatric Gastroenterology Chang Gung Memorial Hospital Taoyuan Linkou Taiwan; ^7^ Division of Pediatric Infectious Diseases, Department of Pediatrics Chang Gung Memorial Hospital Taoyuan Linkou Taiwan; ^8^ Division of Gastroenterology and Hepatology, Department of Internal Medicine New Taipei Municipal Tucheng Hospital New Taipei City Tucheng Taiwan; ^9^ Biostatistical Section, Clinical Trial Center Chang Gung Memorial Hospital Taoyuan Linkou Taiwan

**Keywords:** clinical outcomes, *Clostridioides difficile* infection, fecal microbiota transplantation, inflammatory bowel disease, risk factors

## Abstract

*Clostridioides difficile* infection (CDI) worsens the prognosis of patients with inflammatory bowel disease (IBD). This retrospective cohort study aimed to evaluate the risk factors, clinical manifestations, and outcomes of CDI in hospitalized patients with IBD, including those with toxin A/B results between April 2007 and April 2021. Patients were classified into the CDI and control groups. Patients with IBD and recurrent or refractory CDI underwent fecal microbiota transplantation (FMT). A total of 144 inpatients with IBD—45 in the CDI group and 99 in the control group—were analyzed. The incidence of CDI in inpatients with IBD was 31%. The Risk factors for CDI included longer IBD duration, biological therapy failure, and biological use. More patients in the CDI group presented with abdominal pain (77.8% vs. 55.6%, *p* = 0.011). In the antibiotic treatment‐only group, the symptom improvement rate was 60.7% (17/28), the microbiological cure rate was 89.3% (25/28), and the overall success rate was 71.4% (20/28). After antibiotic treatment and FMT, 71.4% (10/14) of the patients tested negative for CDI, and 64.3% (9/14) had improved clinical symptoms. CDI led to more hospitalizations (median two times [range 0–12] vs. median one time [range 0–19], *p* = 0.008), a lower steroid‐free remission rate (46.7% vs. 67.7%, *p* = 0.017), and higher Mayo scores (median 5 points [range 2–12] vs. median 3 points [range 0–12]). Patients who received FMT had fewer hospitalizations and fewer IBD‐related complications during follow‐up than those who received antibiotics alone. FMT should be considered in patients with IBD with refractory or recurrent CDI to improve clinical outcomes.

## 1 Introduction

*Clostridioides difficile*, a Gram‐positive spore‐forming anaerobe, is the leading cause of nosocomial diarrhea [1]. Patients with inflammatory bowel disease (IBD) have a 4.8‐fold greater risk of *C. difficile* infection (CDI) than those without IBD [2]. It leads to higher risks of acute flares, escalation in medical therapy, colectomy, and mortality in patients with IBD, especially ulcerative colitis (UC) [3, 4, 5, 6]. However, some studies have shown that CDI does not worsen short‐term clinical outcomes, including colon perforation, megacolon, colectomy, shock, respiratory failure, or mortality in IBD within 3 months [7, 8].

Patients with IBD have a higher risk of developing recurrent CDI. Fecal microbiota transplantation (FMT) can achieve a 91% successful eradication rate and a 79%–91% success rate in preventing recurrent CDI [9, 10, 11, 12, 13, 14]. The American College of Gastroenterology and the American Gastroenterological Association clinical guidelines suggest the use of fecal microbiota–based therapies upon completion of standard‐of‐care antibiotics over no fecal microbiota–based therapies in recurrent CDI [15, 16]. Additionally, the first international Rome consensus conference recommended FMT as a treatment option for both mild and severe recurrent or refractory CDI in patients with IBD [17]. However, the therapeutic effect of FMT on IBD‐related outcomes and complications remains unknown. This retrospective study aimed to analyze the risk factors, clinical characteristics, and impact of CDI in inpatients with IBD and to evaluate the role of FMT in this group.

## 2 Materials and Methods

### 2.1 Patients

In this retrospective cohort study, we enrolled hospitalized IBD patients with toxin A/B results for *C. difficile* at a medical center between April 2007 and April 2021. Patients with positive toxin A/B results were enrolled in the CDI group, while the others belonged to the control group. FMT has been used to treat refractory or recurrent CDI. In our study, we utilized a single‐donor, single‐session FMT approach. The procedure was performed via ileocolonoscopy, with 250 mL of microbiota suspension infused directly into the terminal ileum or cecum. No premedications, including antibiotics, were administered prior to the FMT procedure. Donor specimens were sourced from the fecal bank at the Chang Gung Microbiota Therapy Center. Risk factors, clinical manifestations, and outcomes were analyzed. Furthermore, we compared the outcomes of patients who received different treatments.

### 2.2 Data Collection

The medical records of enrolled patients were reviewed for data on age, gender, type of IBD, disease duration, medication, CDI diagnostic date (the date of toxin A/B result), body mass index (BMI), underlying diseases, clinical manifestations, Mayo score, Crohn's Disease Activity Index (CDAI), white blood cell count, hemoglobin, C‐reactive protein, and albumin levels, IBD‐related complications (stricture, perforation, abscess, fistula, colon cancer, and IBD‐related surgery), treatments for CDI (metronidazole, vancomycin, and FMT), therapeutic response, clinical remission, clinical steroid‐free remission, and death or last follow‐up.

### 2.3 Definitions

Microbiological cure means CD toxin and culture were negative within 3 months of treatment. Symptom improvement indicates improved diarrhea and abdominal and bloody stool. Successful treatment is defined as both microbiological cure and symptom improvement. In this study, symptom improvement was evaluated using the Partial Mayo Score for UC and the Crohn's Disease Activity Index (CDAI) for Crohn's disease (CD). For UC, clinical response was defined as a reduction of at least 2 points and ≥ 30% in the Partial Mayo Score from baseline, accompanied by a decrease of at least 1 point in the rectal bleeding subscore or a rectal bleeding score of 0 or 1. For CD, clinical response was defined as a reduction of ≥ 100 points from the baseline CDAI score, with a CDAI score below 150 indicating remission. Refractory CDI is defined as the persistence of symptoms despite appropriate antibiotic therapy for 7–14 days. Recurrence of CDI is defined as recurrent diarrhea accompanied by laboratory confirmation of positive *C. difficile* toxin A/B tests after the completion of a prior successful treatment course.

### 2.4 Statistical Analyses

Numerical data were presented as medians (interquartile range, IQR), while categorical data were presented as absolute numbers and percentages. We used the chi‐square or Fisher's exact test for categorical data and the Mann–Whitney U test for continuous variables. Statistical significance was set at *p* < 0.05. All statistical calculations were performed using SPSS version 22.0 (Armonk, NY, IBM Corp.).

## 3 Results

### 3.1 Baseline Characteristics, Risk Factors, and Outcomes of Hospitalized IBD Patients With *Clostridioides difficile* Infection

A total of 144 inpatients with IBD (45 in CDI group and 99 in control group) were enrolled in the analysis. The median follow‐up duration was 15.5 months. The incidence of CDI in inpatients was 31% (CD, 29%; UC, 34%). The risk factors for CDI include longer IBD duration, biological failure, and biological use. More patients presented with abdominal pain in the CDI group (77.8% vs. 55.6%, *p* = 0.011), especially in Crohn's disease. The other baseline characteristics of the *C. difficile* and control groups are shown in Table 1. Patients in the CDI group received antibiotics as the first‐line treatment, and FMT was performed only for patients with refractory or recurrent CDI. In the antibiotic treatment‐only group, the symptom improvement rate was 60.7%, the microbiological cure rate was 89.3%, and the overall success rate was 71.4%. After receiving antibiotic treatment followed by FMT (14 cases), 71.4% of patients achieved microbiological cure, 64.3% experienced an improvement in clinical symptoms, and the overall success rate was 50%. Regarding clinical outcomes, CDI led to more hospitalizations (median two times [range 0–12 times] vs. median 1 time [range 0–19 times], *p* = 0.008), a lower steroid‐free remission rate (46.7% vs. 67.7%, *p* = 0.017), and a higher Mayo score (median five points [range 2–12 points] vs. median three points [range 0–12 points]). The laboratory data and other clinical outcomes are presented in Table 2.

**TABLE 1:** Baseline characteristics in *Clostridioides difficile*—infected and control groups.

Characteristics	IBD (*n* = 144)	CDI (*n* = 45)	Control (*n* = 99)	*p*	CD (*n* = 79)	CDI (*n* = 23)	Control (*n* = 56)	*p*	UC (*n* = 65)	CDI (*n* = 22)	Control (*n* = 43)	p
Crohn's disease	79 (54.9%)	23 (51.1%)	56 (56.6%)	0.542								
Gender (Male)	89 (61.8%)	27 (60%)	62 (62.6%)	0.764	54 (68.4%)	16 (69.6%)	38 (67.9%)	0.882	35 (53.8%)	11 (50%)	24 (55.8%)	0.656
Age (years)	41.7 (3.4, 78.4)	41 (20.6, 78.4)	41.8 (3.4, 75.6)	0.836	36.7 (3.4, 78.4)	37 (26.2, 78.4)	35.6 (3.4, 75.6)	0.553	44.7 (18.3, 74.1)	44.2 (20.6, 70.7)	44.8 (18.3, 74.1)	0.917
IBD duration (months)	1.4 (0, 228)	6 (0, 144)	0.4 (0, 228)	0.023*	0.8 (0, 228)	3.1 (0, 96)	0.3 (0, 228)	0.103	3.8 (0, 216)	8.2 (0, 144)	0.4 (0, 216)	0.123
Symptoms												
Bloody stool	86 (59.7%)	29 (64.4%)	57 (57.6%)	0.436	32 (40.5%)	10 (43.5%)	22 (39.3%)	0.730	54 (83.1%)	19 (86.4%)	35 (81.4%)	0.737
Diarrhea	88 (61.1%)	28 (62.2%)	60 (60.6%)	0.854	38 (48.1%)	12 (52.2%)	26 (46.4%)	0.642	50 (76.9%)	16 (72.7%)	34 (79.1%)	0.566
Pain	90 (62.5%)	35 (77.8%)	55 (55.6%)	0.011*	54 (68.4%)	21 (91.3%)	33 (58.9%)	0.005*	36 (55.4%)	14 (63.6%)	22 (51.2%)	0.338
Fever	24 (16.7%)	7 (15.6%)	17 (17.2%)	0.809	13 (16.5%)	4 (17.4%)	9 (16.1%)	1.000	11 (16.9%)	3 (13.6%)	8 (18.6%)	0.737
BMI	21.4 (13.6, 36.1)	21.4 (13.6, 29.8)	21.5 (14.1, 36.1)	0.860	21.4 (14.1, 36.1)	21.7 (14.2, 29.8)	21.3 (14.1, 36.1)	0.925	21.4 (13.6, 30.1)	21.1 (13.6, 29)	21.9 (14.5, 30.1)	0.656
Baseline complications												
Stricture	27 (18.8%)	4 (8.9%)	23 (23.2%)	0.041*	23 (29.1%)	3 (13%)	20 (35.7%)	0.044*	4 (6.2%)	1 (4.5%)	3 (7%)	1.000
Perforation	3 (2.1%)	0 (0%)	3 (3%)	0.552	2 (2.5%)	0 (0%)	2 (3.6%)	1.000	1 (1.5%)	0 (0%)	1 (2.3%)	1.000
Abscess	14 (9.7%)	3 (6.7%)	11 (11.1%)	0.549	13 (16.5%)	3 (13%)	10 (17.9%)	0.746	1 (1.5%)	0 (0%)	1 (2.3%)	1.000
Fistula	16 (11.1%)	5 (11.1%)	11 (11.1%)	1.000	15 (19%)	4 (17.4%)	11 (19.6%)	1.000	1 (1.5%)	1 (4.5%)	0 (0%)	0.338
Colon cancer	1 (0.7%)	0 (0%)	1 (1%)	1.000	1 (1.3%)	0 (0%)	1 (1.8%)	1.000	0 (0%)	0 (0%)	0 (0%)	—
IBD surgery	28 (19.4%)	8 (17.8%)	20 (20.2%)	0.733	24 (30.4%)	7 (30.4%)	17 (30.4%)	0.995	4 (6.2%)	1 (4.5%)	3 (7%)	1.000
IBD complications	48 (33.3%)	13 (28.9%)	35 (35.4%)	0.446	40 (50.6%)	11 (47.8%)	29 (51.8%)	0.749	8 (12.3%)	2 (9.1%)	6 (14%)	0.706
IBD medication												
Biological failure	38 (26.4%)	17 (37.8%)	21 (21.2%)	0.037*	22 (27.8%)	9 (39.1%)	13 (23.2%)	0.152	16 (24.6%)	8 (36.4%)	8 (18.6%)	0.116
Biologics user	27 (18.8%)	13 (28.9%)	14 (14.1%)	0.036*	16 (20.3%)	8 (34.8%)	8 (14.3%)	0.062	11 (16.9%)	5 (22.7%)	6 (14%)	0.487
Infliximab	1 (0.7%)	1 (2.2%)	0 (0%)		1 (1.3%)	1 (4.3%)	0 (0%)		0 (0%)	0 (0%)	0 (0%)	
Adalimumab	13 (9%)	9 (20%)	4 (4%)		8 (10.1%)	5 (21.7%)	3 (5.4%)		5 (7.7%)	4 (18.2%)	1 (2.3%)	
Vedolizumab	12 (8.3%)	3 (6.7%)	9 (9.1%)		6 (7.6%)	2 (8.7%)	4 (7.1%)		6 (9.2%)	1 (4.5%)	5 (11.6%)	
Ustekinumab	1 (0.7%)	0 (0%)	1 (1%)		1 (1.3%)	0 (0%)	1 (1.8%)		0 (0%)	0 (0%)	0 (0%)	
5‐ASA	103 (71.5%)	31 (68.9%)	72 (72.7%)	0.636	55 (69.6%)	17 (73.9%)	38 (67.9%)	0.595	48 (73.8%)	14 (63.6%)	34 (79.1%)	0.180
Oral prednisolone	78 (54.2%)	29 (64.4%)	49 (49.5%)	0.095	43 (54.4%)	15 (65.2%)	28 (50%)	0.217	35 (53.8%)	14 (63.6%)	21 (48.8%)	0.257
Dosage (mg/day)	5 (0, 40)	10 (0, 40)	0 (0, 40)	0.158	5 (0, 40)	5 (0, 30)	1.3 (0, 40)	0.243	5 (0, 40)	10 (0, 40)	0 (0, 40)	0.450
Azathioprine	32 (22.2%)	11 (24.4%)	21 (21.2%)	0.665	25 (31.6%)	7 (30.4%)	18 (32.1%)	0.882	7 (10.8%)	4 (18.2%)	3 (7%)	0.215
Antibiotics	64 (44.4%)	20 (44.4%)	44 (44.4%)	1.000	35 (44.3%)	12 (52.2%)	23 (41.1%)	0.367	29 (44.6%)	8 (36.4%)	21 (48.8%)	0.338
Mesalazine enema	34 (23.6%)	12 (26.7%)	22 (22.2%)	0.561	3 (3.8%)	1 (4.3%)	2 (3.6%)	1.000	31 (47.7%)	11 (50%)	20 (46.5%)	0.790
Steroid enema	7 (4.9%)	3 (6.7%)	4 (4%)	0.678	0 (0%)	0 (0%)	0 (0%)	—	7 (10.8%)	3 (13.6%)	4 (9.3%)	0.681

*Note*: Age, IBD duration, BMI, steroid dosage, and steroid dose change are presented as median (IQR). The remaining data are presented as number (percentage).

Abbreviations: 5‐ASA, 5‐aminosalicylic acid; ESRD, end‐stage renal disease; IBD, inflammatory bowel disease; IQR, interquartile range.

* *p* < 0.05.

**TABLE 2:** Laboratory data and clinical outcomes in *Clostridioides difficile*—infected and control groups.

Characteristics	IBD (*n* = 144)	CDI (*n* = 45)	Control (*n* = 99)	*p*		CD (*n* = 79)	CDI (*n* = 23)	Control (*n* = 56)	*p*	UC (*n* = 65)	CDI (*n* = 22)	Control (*n* = 43)	*p*
Laboratory data													
WBC(1000/μL)	7.7 (2.1, 23.7)	7.8 (3.2, 20.1)	7.7 (2.1, 23.7)	0.819		7.7 (2.1, 20.1)	7.9 (4.7, 20.1)	7.6 (2.1, 18.2)	0.651	8 (3.2, 23.7)	7.7 (3.2, 15.3)	8.3 (4.2, 23.7)	0.453
Hb (g/dL)	11.8 (6.5, 36.8)	11.7 (7.4, 15.7)	11.9 (6.5, 36.8)	0.931		11.3 (6.5, 36.8)	11.4 (7.7, 15.5)	10.8 (6.5, 36.8)	0.639	12.3 (7.4, 15.7)	12.3 (7.4, 15.7)	12.3 (8.2, 15.7)	0.694
CRP (mg/dL)	13.5 (0.2, 273.8)	8.9 (0.2, 159.7)	17.1 (0.2, 273.8)	0.290		18.7 (0.2, 152.3)	11.8 (0.2, 142.9)	23.7 (0.2, 152.3)	0.467	7.9 (0.2, 273.8)	3.6 (0.2, 159.7)	8.7 (0.2, 273.8)	0.480
Albumin (g/dL)	3.6 (1.4, 4.8)	3.8 (2.3, 4.7)	3.5 (1.4, 4.8)	0.438		3.5 (1.8, 4.7)	3.6 (3.2, 4.7)	3.5 (1.8, 4.4)	0.216	4 (1.4, 4.8)	4 (2.3, 4.3)	3.7 (1.4, 4.8)	0.574
CMV co‐infection	15 (13%)	5 (11.4%)	10 (14.1%)	0.674		4 (6.8%)	1 (4.5%)	3 (8.1%)	1.000	11 (19.6%)	4 (18.2%)	7 (20.6%)	1.000
Treatments	—	42 (93.3%)	—	—		—	20 (87%)	—	—	—	22 (100%)	—	—
Metronidazole (po)	—	41 (91.1%)	—	—		—	20 (87%)	—	—	—	21 (95.5%)	—	—
Vancomycin (po)	—	3 (6.7%)	—	—		—	1 (4.3%)	—	—	—	2 (9.1%)	—	—
FMT	—	14 (31.1%)	—	—		—	3 (13%)	—	—	—	11 (50%)	—	—
Treatment results													
Symptom improve	—	26 (61.9%)	—	—		—	11 (55%)	—	—	—	15 (68.2%)	—	—
Microbiological cure	—	35 (83.3%)	—	—		—	18 (90%)	—	—	—	17 (77.3%)	—	—
Outcomes (overall)													
Steroid dose change	0 (−40, 20)	−2.5 (−40, 15)	0 (−40, 20)	0.742		0 (−40, 20)	−2.5 (−30, 10)	0 (−40, 20)	0.845	0 (−40, 15)	−5 (−40, 15)	0 (−40, 5)	0.508
Hospitalization times	1 (0, 19)	2 (0, 12)	1 (0, 19)	0.008*		1 (0, 19)	3 (0, 12)	1 (0, 19)	0.019*	1 (0, 7)	2 (0, 7)	1 (0, 7)	0.133
Clinical remission	102 (70.8%)	28 (62.2%)	74 (74.7%)	0.125		53 (67.1%)	12 (52.2%)	41 (73.2%)	0.071	49 (75.4%)	16 (72.7%)	33 (76.7%)	0.722
SFCR	88 (61.1%)	21 (46.7%)	67 (67.7%)	0.017*		45 (57%)	9 (39.1%)	36 (64.3%)	0.04*	43 (66.2%)	12 (54.5%)	31 (72.1%)	0.157
CDAI	139 (0, 464.6)	145.4 (44.7, 464.6)	137 (0, 386.7)	0.620		139 (0, 464.6)	145.4 (44.7, 464.6)	137 (0, 386.7)	0.620	—	—	—	—
Mayo score	3 (0, 12)	5 (2, 12)	3 (0, 12)	0.039*		—	—	—	—	3 (0, 12)	5 (2, 12)	3 (0, 12)	0.039*
BMI change	0.4 (−30.1, 8.8)	1 (−5.5, 7.5)	0.2 (−30.1, 8.8)	0.093		0.3 (−22.5, 8.8)	0.6 (−5.5, 4.8)	0.3 (−22.5, 8.8)	0.564	0.4 (−30.1, 7.5)	1.1 (−2.9, 7.5)	0.1 (−30.1, 3.8)	0.064
Stricture	40 (27.8%)	10 (22.2%)	30 (30.3%)	0.316		32 (40.5%)	8 (34.8%)	24 (42.9%)	0.507	8 (12.3%)	2 (9.1%)	6 (14.0%)	0.706
Perforation	3 (2.1%)	0 (0%)	3 (3%)	0.552		2 (2.5%)	0 (0%)	2 (3.6%)	1.000	1 (1.5%)	0 (0%)	1 (2.3%)	1.000
Abscess	16 (11.1%)	3 (6.7%)	13 (13.1%)	0.253		15 (19%)	3 (13%)	12 (21.4%)	0.533	1 (1.5%)	0 (0%)	1 (2.3%)	1.000
Fistula	20 (13.9%)	6 (13.3%)	14 (14.1%)	0.897		19 (24.1%)	5 (21.7%)	14 (25%)	0.758	1 (1.5%)	1 (4.5%)	0 (0%)	0.338
Colon cancer	3 (2.1%)	1 (2.2%)	2 (2%)	1.000		1 (1.3%)	0 (0%)	1 (1.8%)	1.000	2 (3.1%)	1 (4.5%)	1 (2.3%)	1.000
IBD surgery	38 (26.4%)	13 (28.9%)	25 (25.3%)	0.646		29 (36.7%)	8 (34.8%)	21 (37.5%)	0.820	9 (13.8%)	5 (22.7%)	4 (9.3%)	0.253
IBD complications	64 (44.4%)	20 (44.4%)	44 (44.4%)	1.000		49 (62%)	14 (60.9%)	35 (62.5%)	0.892	15 (23.1%)	6 (27.3%)	9 (20.9%)	0.566
Death	4 (2.8%)	1 (2.2%)	3 (3%)	1.000		3 (3.8%)	0 (0%)	3 (5.4%)	0.552	1 (1.5%)	1 (4.5%)	0 (0%)	0.338
Follow—up duration (months)	15.5 (0.1, 138.7)	14.8 (0.6, 66.3)	16.1 (0.1, 138.7)	0.668		16.3 (0.8, 98.5)	14.8 (0.8, 66.3)	16.5 (1.1, 98.5)	0.718	13.7 (0.1, 138.7)	14.2 (0.6, 64.2)	13.7 (0.1, 138.7)	0.873

*Note*: Hospitalization, CDAI, Mayo score, BMI change, and follow—up duration are presented as median (IQR). The remaining data are presented as number (percentage).

Abbreviations: CDAI, Crohn's disease activity index; CMV, cytomegalovirus; IBD, inflammatory bowel disease; IHC, immunohistochemistry; IQR, interquartile range; SFCR, steroid free clinical remission; WBC, white blood cell.

* *p* < 0.05.

### 3.2 The Impact of Treatments for *Clostridioides difficile* Infection

Compared with the control group, the patients with microbiological cure after treatment still had more hospitalizations (median three times [range 0–12 times] vs. median one time [range 0–19 times]) and higher Mayo scores (median 5.5 points [range 2–12 points] vs. median three points [range 0–12 points]) at the end of the study. Other details are listed in Table 3. In patients receiving FMT, ulcerative colitis (78.6%) was predominant, and the opposite was observed in patients receiving antibiotics alone (Crohn's disease, 60.7%) (*p* = 0.016). Compared with patients receiving antibiotics alone, those receiving FMT had fewer hospitalizations (median one time [range 0–5 times] vs. median four times [range 0–12 times]) and fewer IBD‐related complications (21.4% vs. 53.6%) during the follow‐up. The follow‐up period was 9.3 months for the FMT group and 17.9 months for the antibiotic treatment‐only group. The overall follow‐up periods were similar since the initiation of antibiotic use. In the FMT group, we observed no treatment failures; however, four patients experienced a single recurrence of CDI during the follow‐up period. The additional details are provided in Table 4.

**TABLE 3:** Baseline characteristics and clinical outcomes in *Clostridioides difficile*—infected patients with microbiological cure after treatment and control groups.

Characteristics	Microbiological cure (*n* = 35)	Control (*n* = 99)	*p*
Inflammatory bowel disease			0.599
Crohn's disease	18 (51.4%)	56 (56.6%)	
Ulcerative colitis	17 (48.6%)	43 (43.4%)	
Gender			
Male	21 (60%)	62 (62.6%)	0.783
Age (years)	39.1 (20.6, 78.4)	41.8 (3.4, 75.6)	0.899
IBD duration (months)	4.2 (0, 144)	0.4 (0, 228)	0.111
Outcomes (overall)			
Steroid dose change	−5 (−40, 15)	0 (−40, 20)	0.645
Hospitalization times	3 (0, 12)	1 (0, 19)	< 0.001*
Clinical remission	22 (62.9%)	74 (74.7%)	0.180
CDAI	147.5 (44.7, 464.6)	137 (0, 386.7)	0.556
Mayo score	5.5 (2, 12)	3 (0, 12)	0.035*
BMI change	1.1 (−5.5, 5.8)	0.2 (−30.1, 8.8)	0.088
Stricture	6 (17.1%)	30 (30.3%)	0.131
Perforation	0 (0%)	3 (3%)	0.567
Abscess	2 (5.7%)	13 (13.1%)	0.352
Fistula	4 (11.4%)	14 (14.1%)	0.781
Colon cancer	1 (2.9%)	2 (2%)	1.000
IBD surgery	10 (28.6%)	25 (25.3%)	0.701
IBD complications	16 (45.7%)	44 (44.4%)	0.897
Death	1 (2.9%)	3 (3%)	1.000

*Note*: Mann—Whitney U test on continuous variable and Chi—square and Fisher's exact test on categorical data were used.

*p < 0.05.

**TABLE 4:** Baseline characteristics and clinical outcomes in FMT and antibiotic treatment—only groups.

Characteristics	FMT (*n* = 14)	Antibiotic treatment—only (*n* = 28)	*p*
Inflammatory bowel disease			0.016*
Crohn's disease	3 (21.4%)	17 (60.7%)	
Ulcerative colitis	11 (78.6%)	11 (39.3%)	
Gender			
Male	9 (64.3%)	16 (57.1%)	0.657
Age (years)	44.6 (20.6, 70.7)	38.9 (26.2, 78.4)	0.947
IBD duration (months)	11.6 (0, 84)	4.2 (0, 144)	0.762
Treatment results			
Symptom improvement	9 (64.3%)	17 (60.7%)	0.822
Microbiological cure	10 (71.4%)	25 (89.3%)	0.197
Successful treatment	7 (50%)	20 (71.4%)	0.172
Outcomes (overall)			
Steroid dose change	0 (−20, 0)	−6.3 (−40, 15)	0.683
Hospitalization times	1 (0, 5)	4 (0, 12)	0.023*
Clinical remission	9 (64.3%)	17 (60.7%)	0.822
CDAI	187.6 (67.9, 307.2)	147.5 (44.7, 464.6)	0.941
Mayo score	7 (2, 12)	4.5 (2, 12)	0.393
BMI change	0.7 (−1, 7.5)	1.1 (−5.5, 5.8)	0.927
Stricture	1 (7.1%)	7 (25%)	0.233
Perforation	0 (0%)	0 (0%)	—
Abscess	0 (0%)	3 (10.7%)	0.539
Fistula	2 (14.3%)	3 (10.7%)	1.000
Colon cancer	0 (0%)	1 (3.6%)	1.000
IBD surgery	2 (14.3%)	10 (35.7%)	0.277
IBD complications	3 (21.4%)	15 (53.6%)	0.047*
Death	0 (0%)	1 (3.6%)	1.000

*Note*: Mann—Whitney U test on continuous variable and Chi—square and Fisher's exact test on categorical data were used.

* *p* < 0.05.

## 4 Discussion

Reported risk factors for CDI in IBD include the use of antibiotics, steroids, immunomodulators, biologics, nonsteroidal anti‐inflammatory drugs, and proton pump inhibitors [18]. Other factors included recent hospitalization, history of colectomy, ileal anal‐pouch anastomosis, CMV infection, disease severity, disease extension, and genetic factors [18]. In our study, we identified the IBD duration and biological failure as new risk factors. Previous studies reported that the incidence of CDI in adult CD and UC inpatients was 1%–7.7% and 2.8%–11.1%, respectively [19, 20, 21, 22, 23, 24, 25]. In our study, the incidence of CDI was 29% in inpatients with CD and 34% in inpatients with UC. The incidence observed in our study was significantly higher than that reported in Western countries and one possible explanation for this is our routine practice of testing for *C. difficile* toxin in IBD patients admitted for refractory disease or acute flare‐ups and the possibility of false‐positive results. The findings suggested that the prevalence of CDI in IBD inpatients has likely been underestimated. To improve patient outcomes, routine screening for CDI should be considered for IBD patients presenting with refractory disease or acute flare‐ups. CDI leads to poor prognosis in IBD patients, including higher colectomy rates, therapeutic escalation, long‐term hospitalizations, increased readmission rates, increased in‐hospital expenditures, and even higher mortality [18]. In this study, we found that CDI resulted in higher readmission rates, poor steroid‐free clinical remission rates in CD, and higher Mayo scores in UC at the end of the study. Even if the patients had a microbiological cure, they still had higher readmission rates and Mayo scores because *C. difficile* also indicates dysbiosis [26]. FMT is effective for the treatment of recurrent CDI n in patients without IBD, as well as in patients with UC and CD [11, 17, 27, 28]. FMT could treat refractory or recurrent CDI and rebuild the healthy microbiota of the gut, such that it may contribute to fewer overall IBD‐related complications compared to antibiotic treatment alone.

According to the European Crohn's and Colitis Organization (ECCO) guidelines, oral vancomycin, and fidaxomicin are considered equally effective for the treatment of non‐severe CDI, with a recommended duration of 10 days. For severe CDI, it is recommended to combine intravenous metronidazole with oral vancomycin for the same duration. However, the use of metronidazole as a standalone therapy is no longer guideline‐compliant due to its relatively lower efficacy and higher risk of side effects. In our study, the majority of patients received metronidazole, while only a small number were treated with vancomycin. This treatment strategy reflects historical clinical practices and likely contributed to the lower cure rate observed with antibiotic therapy. Management of recurrent CDI includes options such as oral vancomycin, fidaxomicin, FMT, and bezlotoxumab [29]. However, there is insufficient evidence to support FMT as a treatment for the first episode of CDI in patients with IBD. A systematic review demonstrated that FMT significantly increases the resolution of recurrent CDI compared to antibiotic therapy [30]. Furthermore, a randomized trial of patients with recurrent CDI revealed that combining FMT with a 10‐day course of fidaxomicin or vancomycin was superior to using either antibiotic alone in achieving clinical and microbiological resolution [31]. According to the latest 2024 Rome Consensus, FMT is now recommended as a treatment option for both mild and severe recurrent or refractory CDI in patients with IBD [17]. Real‐world studies further support FMT as an effective, safe, and rational therapeutic alternative for managing recurrent CDI in patients with underlying IBD [32, 33]. This aligns with our findings, which highlight the superior clinical outcomes of FMT, including reduced hospitalization rates and fewer IBD‐related complications, compared to antibiotic treatment alone. However, there is currently no clear benefit to combining FMT with bezlotoxumab compared to FMT alone for treating recurrent CDI in IBD patients [34].

In this study, the symptomatic improvement, microbiological cure, and successful treatment rates were 64.3%, 71.4%, and 50%, respectively. In a previous prospective study on FMT for refractory or recurrent CDI, a 91% success rate was reported, defined by the resolution of diarrhea and undetectable *C. difficile* toxins [14]. Other studies demonstrated the cure rate around 70% [33, 35]. The variation in success rates may be attributed to the stricter definition of symptomatic improvement used in our study, which considers not only the resolution of diarrhea and a negative *C. difficile* toxin result but also additional symptoms such as abdominal pain and bloody stool. In Table 4, we compare the antibiotic treatment‐only group with the antibiotics‐plus‐FMT group, focusing on the target intervention, FMT. Our primary objective in Table 4 was not to evaluate the success rates of individual interventions in isolation but to examine the clinical outcomes and broader impact of FMT in patients with recurrent or refractory CDI. The limitations of this study include its retrospective design and small sample size. Furthermore, as many patients were referred from local hospitals for FMT, the specific reasons behind the initial selection of antibiotics could not be ascertained. These decisions were likely influenced by individual clinical judgment and the availability of local resources. To address these limitations, a larger, prospective, multicenter study is warranted in the future.

## 5 Conclusions

In this comprehensive study on refractory or recurrent CDI in patients with IBD, the incidence of CDI was much higher in Taiwan than in Western countries. Overuse of antibiotics in patients with IBD should be avoided because CDI can lead to a poor prognosis. FMT was not only effective in eradicating *C. difficile* but also decreased the readmission rate and IBD‐related complications.

## Disclosure

The abstract of this paper was presented at the 2023 European Crohn's and Colitis Organization (ECCO) as a poster presentation with interim findings. The poster's abstract was published in ‘Poster Abstracts’ in Journal of Crohn's and Colitis, Volume 17, Issue Supplement_1, February 2023, Page i582, https://doi.org/10.1093/ecco‐jcc/jjac190.0582.

## Ethics Statement

The study was approved by the Institutional Review Board (IRB) of the Chang Gung Memorial Hospital, Linkou (approval document No. 202101234B0: “Clinical presentations and outcome of cytomegalovirus, herpes simplex virus, Epstein‐Barr virus, and *Clostridioides* infection”) for the period 28 July 2021–27 July 2022. This study is retrospective in nature, and the ethics committee approved a consent waiver for it. The study protocol conforms to the ethical guidelines of the 1975 Declaration of Helsinki. This work was also supported by Ministry of Health and Welfare (Taiwan), MOHW113‐TDU‐B‐212‐114010.

## Conflicts of Interest

The authors declare no conflicts of interest.

## Data Availability

The data that support the findings of this study are available on request from the corresponding author. The data are not publicly available due to privacy or ethical restrictions.
